# Effect of strength training with additional acupuncture on balance, ankle sensation, and isokinetic muscle strength in chronic ankle instability among college students

**DOI:** 10.3389/fphys.2024.1324924

**Published:** 2024-04-05

**Authors:** Shuwan Chang, Yajun Tan, Liang Cheng, Liping Zhou, Bingcheng Wang, Heng Liu

**Affiliations:** ^1^ School of Sports Medicine and Health, Chengdu Sport University, Chengdu, China; ^2^ Department of Sports and Human Science, Sichuan Sports College, Chengdu, China; ^3^ Sport Hospital, Chengdu Sport University, Chengdu, China; ^4^ Department of General Practice, Affiliated Jinling Hospital, Medical School of Nanjing University, Nanjing, China; ^5^ College of Physical Education, Chongqing University, Chongqing, China

**Keywords:** chronic ankle instability, muscle strength, acupuncture, posture control, balance

## Abstract

**Purpose:** The effects of the combination of strength training and acupuncture on chronic ankle instability have not been studied. This study examined effects of strength training combined with acupuncture on balance ability, ankle motion perception, and muscle strength in chronic ankle instability among college students.

**Methods:** Forty-six chronic ankle instability college students were randomly categorized into the experimental group (n = 24, strength training + acupuncture) and the control group (n = 22, strength training) for an 8-week intervention.

**Results:** For the results at 8 weeks, compared with the baseline, in the experimental group, the chronic Ankle Instability Tool (CAIT) score, ankle dorsiflexion, plantar flex, eversion peak torque (60°/s), and plantar flex peak torque (180°/s) increased by 13.7%, 39.4%, 13.7%, 14.2%, and 12.3%, respectively. Dorsiflexion, plantar flexion, inversion, and eversion kinesthetic sensation test angles decreased by 17.4%, 20.6%, 15.0%, and 17.2%, respectively. Anterior–posterior and medial–lateral displacement, and anterior–posterior and medial–lateral velocity decreased by 28.9%, 31.6%, 33.3%, and 12.4%, respectively. Anterior–posterior and medial–lateral displacement, and anterior–posterior and medial–lateral mean velocity decreased by 28.9%, 31.6%, 33.3%, and 12.4%, respectively. In the control group, the Cumberland Ankle Instability Tool score and the ankle dorsiflexion peak torque (60°/s) increased by 13.8% and 17.9%, respectively. The inversion kinesthetic sensation test angle decreased by 15.2%, whereas anterior–posterior and medial–lateral displacement, and anterior–posterior and medial–lateral mean velocity decreased by 17.1%, 29.4%, 12.3%, and 16.8%, respectively. 2) For the comparison between the groups after 8 weeks, the values of ankle dorsiflexion and plantar flex peak torque (60°/s) in the experimental group were greater than those in the control group. The values of ankle plantar flex kinesthetic sensation test angle, the anterior–posterior displacement, and anterior–posterior mean velocity in the experimental group were lower than those in the control group.

**Conclusion:** Acupuncture treatment in conjunction with muscle strength training can further improve the balance ability of anterior–posterior, ankle dorsiflexion, and plantar flex strength and plantar flex motion perception in chronic ankle instability participants.

## 1 Introduction

Chronic ankle instability (CAI) refers to the clinical phenomenon of repeated ankle sprains caused by ankle instability, including ankle pain, weakness, reduced range of motion, self-reported decreased function, and other symptoms (Lin et al., 2021; Li et al., 2023). CAI can cause a decrease in human postural control (Lee et al., 2022), such as balance (Lee and Choi, 2019; Liu et al., 2023), decreased ankle muscle strength (Khalaj et al., 2020; Kaminski and Hartsell, 2002), ankle proprioception loss (Ma et al., 2021; Han et al., 2022), and increased lower-limb muscle nerve response time (Levin et al., 2015; Levin et al., 2015). Among patients with their first ankle sprain, approximately 30.3% (206 cases) experienced ankle resprain within 5 years (Mailuhu et al., 2018). Repeated sprains caused by CAI result in further damage of soft tissues such as ligaments and tendons near the ankle (Guillo et al., 2013; Hertel and Corbett, 2019), and even induce ankle osteoarthritis (Herzog et al., 2019; Kim et al., 2020). Therefore, effective intervention for CAI population is necessary.

CAI is associated with muscle strength deficiency (Herzog et al., 2019; Kim et al., 2020), and strength training can be used to prevent ankle resprain (Janssen et al., 2011). Current strength training in the CAI population focuses on hip and knee (Negahban et al., 2013; Lu et al., 2022), ankle (Khalaj et al., 2020; Kaminski and Hartsell, 2002), and the core region (Dastmanesh, et al., 2012; Alizamani et al., 2023). Among them, ankle strength mainly uses different forms of resistance training, but the effect of ankle strength training alone is not good, and it requires other intervention methods such as proprioceptive neuromuscular facilitation (Hall et al., 2015; Hall et al., 2018). Acupuncture, as a Chinese medical treatment, can reduce participants’ pain (stimulate specific acupoints, promote blood circulation and nerve conduction, and reduce pain) (Huang et al., 2023), improve muscle control (adjust muscle tension and coordination) (Zhang et al., 2022), promote tissue repair (accelerate blood circulation and metabolism, promote damaged tissue repair, and regeneration) (de Oliveira and de Freitas, 2016), and improve the nervous system (adjust nerve conduction and regulation) (López et al., 2021). Acupuncture and dry needling (dry acupuncture) are two common treatments that share some similarities in using long, thin needles to stimulate the body to promote self-healing (Zhu and Most, 2016). However, there are some important differences in practice. First, acupuncture is an integral part of traditional Chinese medicine, which has been around for thousands of years in China. It is based on the theory of meridians and the concept of Qi and blood balance, and adjusts the flow of energy within the body by inserting long, thin metal needles into specific acupuncture points. These points are associated with various organs and systems of the body and are believed to influence health status (Zhou et al., 2015). Dry needling, on the other hand, has its origins in modern Western medicine, and its theoretical basis is mainly based on neuromuscular anatomy. The method is usually performed by a trained physiotherapist, orthopedist, or other professional. Dry needling involves inserting long, thin needles directly into stiff, constricted, or sensitive nodules near the surface of the skin to release muscle tension and improve motor function (Zhu and Most, 2016). It should be noted that although both methods involve using similar morphological structures (i.e., metal rods) as tools to exert pressure or stimulate specific areas, the rationale and goals behind them are slightly different. Whereas clock acupuncture emphasizes overall balance and energy flow, dry needling focuses on relieving local problems and improving motor performance (Zhu and Most, 2016). Acupuncture has positive effects in relieving pain (Park et al., 2013) in CAI participants, promoting ankle proprioceptive recovery (Yan et al., 2013; Luan et al., 2023), and increasing the ankle range of motion (Mullins et al., 2021). To the best of our knowledge, additional acupuncture intervention for strength training CAI has not been studied. We clinically treated CAI participants with strength training with concurrent acupuncture. However, its effect on posture control in CAI participants remains unclear.

This study examined effects of strength training combined with acupuncture on balance ability, ankle motion perception, and muscle strength on chronic ankle instability among college students. This study hypothesized that strength training with additional acupuncture further improves balance, ankle kinesthetic sensation, and muscle strength in CAI participants compared to strength training alone.

## 2 Materials and methods

### 2.1 Participants

This study was approved by the Sichuan Sports College Ethics Committee for Human Testing (No: 202301; approval date: 1 January 2023). Based on study results and a prior CAI intervention (Hall et al., 2015), as well as an experimental design of 2 (groups), 2 (number of measurements), and a sample turnover rate of approximately 10%, the required number of participants is at least 44 according to G-power (version 3.1.9.7, Heinrich Heine University, Germany) calculations with an effect size of 0.3, power of 0.8, and α = 0.05.

The inclusion criteria were as follows: age 18–25 years with unilateral CAI and should have passed a health examination. The CAI criteria of the Cumberland Ankle Instability Tool (CAIT) were used for screening (at least one severe ankle sprain, 12 months before the questionnaire survey, pain, swelling and other inflammatory symptoms; could not participate in daily activities for more than 1 day; uncontrolled or sprain or instability in the last 1 year; and CAIT score less than 24 points) (Hiller et al., 2006). The study complied with the Declaration of Helsinki, and all participants signed informed consent forms.

The exclusion criteria were as follows: presence of foot deformity and gait abnormalities; history of lower limb trauma; movement disorder, epilepsy, or cardiovascular disease; and positive ankle anterior drawer test and talus tilt test, excluding structural ankle instability (Lee et al., 2014). Forty-eight (male/female: 28/20) participants with unilateral ankle instability were recruited, and two participants withdrew because of personal reasons. Numbers were randomly assigned, and the participants were categorized into the experimental group (*n* = 24, male/female: 14/10) and the control group (*n* = 22, male/female: 13/9, Table 1). No significant difference was observed in the age, height, and body mass between groups (*p* > 0.05).

**TABLE 1:** Basic information of the study participants.

Group	N (male/female)	Age (y)	Stature (cm)	Body mass (kg)
Experimental group	24 (14/10)	21.6 ± 2.2	172.5 ± 5.8	65.2 ± 7.3
Control group	22 (13/9)	21.4 ± 1.9	171.9 ± 6.2	64.8 ± 7.8

### 2.2 Strength training and acupuncture intervention

The experimental group underwent strength training and acupuncture intervention. The control group only performed the strength training. Intervention duration was 8 weeks, with frequency of 3 times/week. All participants in the experimental group received acupuncture intervention after completing strength training.

**FIGURE 1:**
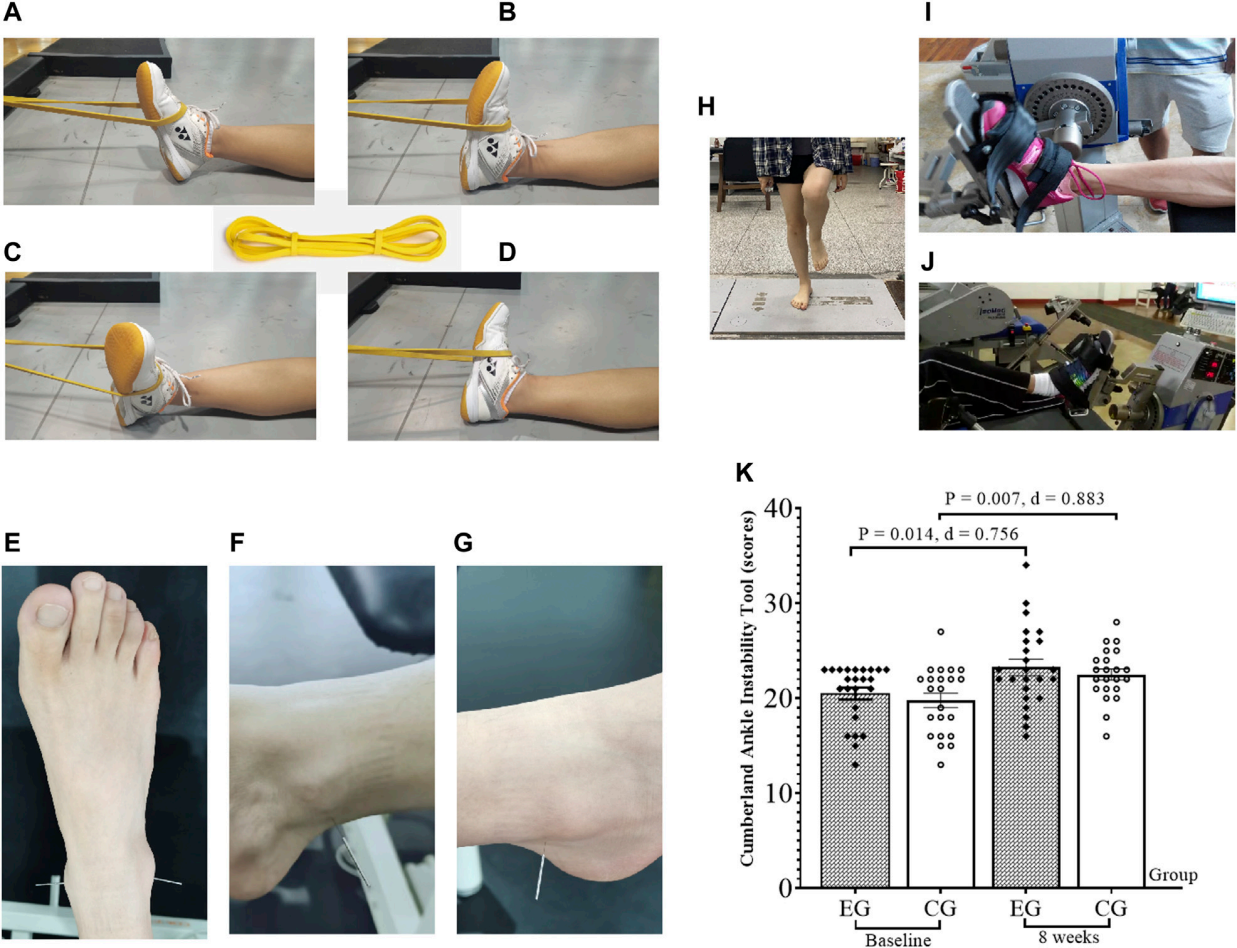
Test the figure and Cumberland Ankle Instability Tool scores. EG, experimental group; CG, control group; ankle strength training **(A–D)**; ankle acupuncture **(F–G)**; balance ability test **(H)**; isokinetic strength test **(I)**; plantar flex and dorsiflexion **(J)**; invert and evert; Cumberland Ankle Instability Tool **(K)**; d, Cohen’s d.

**Strength training:** The participants were positioned flat and conducted resistance training with an elastic belt (elastic belt brand: Joinfit; color: yellow; specifications: 2,080 mm × 4.5 mm × 6.4 mm; and resistance: 5–15 lbs). One end of the elastic band was fixed, and the other end was wound around the metatarsal of the participant; plantar flex, dorsiflexion, and invert and evert of the ankle motion were performed (Figures 1A–D) (Hall et al., 2015). Based on the training protocol, four ankle exercises (plantar flex, dorsiflexion, invert, and evert) were carried out, and five sets of exercises were performed for 10–15 times per group, and rest was permitted for 15–20 s between groups.

**Acupuncture:** Acupuncture was performed at two acupoints (Taixi and Kunlun, Figures 1E) around the ankle of the participants using a filiform needle (manufacturer: Acupro Medical Instruments China, model: 0.25 × 25 mm). Taixi (KI3) (Figures 1F) was located in the rear of the medial malleolus and the depression between the Achilles tendon. This acupoint can promote the movement of blood, relieve the congestion of the foot, and have an analgesic effect. This point can be used for the treatment of medial malleolar injury (Zhu et al., 2015), and Kunlun (BL 60) (Figures 1G) was located in the depression between the posterior lateral malleolar tip and the Achilles tendon; acupuncture at this point has analgesic effect (Zhu et al., 2012). The acupuncture time of both acupoints was the same (approximately 10–15 min). All the participants did not change their previous life conditions, and they had a weekly telephone visit to record their living conditions.

This study involved two measurements (baseline, 8 weeks) for all participants to compare the effect of the intervention for strength training and acupuncture *versus* strength training.

### 2.3 CAIT test

Diagnosis and grading of ankle stability were assessed using nine questions with a test designed by Hiller et al. (2006). Per participant, each ankle was scored separately, and each question was assigned different scores according to the number of options, with the highest unilateral ankle score of 30 points. The standard for unilateral ankle instability was ≤24 points. For Chinese participants, CAIT has a good test–retest reliability (ICC = 0.930) and fine internal consistency (Cronbach’s alpha = 0.845–0.878) (Wang et al., 2021). Participant unstable side (one side) data were selected for analysis in this study.

### 2.4 Balance ability test

Balance test was carried out on the unstable ankle (unilateral) while standing on a three-dimensional measuring table (Kistler, model: 9287B; Figures 1H). The test was carried out for 10 s and repeated three times (1 min) (Wang et al., 2023). Data from three measurements were averaged. The test indicators were the maximum displacement and mean velocity at the pressure center in the anterior–posterior direction and in the medial–lateral direction. Larger data indicate poorer balance ability (Wang et al., 2023).

### 2.5 Ankle isokinetic muscle strength test

All participants were tested with plantar flex, dorsiflexion, and invert and evert (60°/s for five repetitions and 180°/s for five repetitions) with an isokinetic dynamometer (Germany, IsoMed 2000). For plantar and dorsiflexion testing, participants laid supine (Figures 1I, range of motion, 70°; plantar flex exercise, 50°; dorsiflexion motion, 20° when the ankle was at the vertical ground position). For invert and evert testing, participants were seated (Figures 1J, range of motion, 70°; invert motion, 40°; evert motion, 30° when the ankle was at the vertical ground position). Before the testing, participants were allowed to warm up for 10 min. Peak torque (PT) refers to the maximum output torque (Nm) generated by muscle contraction during the whole joint activity, and this parameter indicates the strength quality (Cheng and Jiang, 2020; Cheng et al., 2022).

### 2.6 Ankle kinesthetic sensation test

An isokinetic muscle strength tester (Germany, IsoMed 2000) was used to test the participant’s ankle kinesthetic sensation (Wang et al., 2023). After the test, the angular velocity was set at 0.3°/s. External interference was eliminated by asking the participants to cover their eyes with black cloth, wear headphones, and listen to music. The isokinetic device made the ankle plantar flex, dorsiflexion, and invert and evert.

When participants perceived the ankle movement, they pressed a button (the isokinetic device stopped the movement), and the angle of the joint movement was recorded (also known as the threshold). Each participant underwent three measurements, and the average was obtained; the smaller the angle, the better the kinesthetic sensation.

### 2.7 Statistical analysis

Data were expressed as the mean ± standard deviation by SPSS 20.0. The design involved two groups and two time periods (Cheng et al., 2021). The Shapiro–Wilk test was used to check the normality of the measured data, which would be transformed if it did not conform to the normal distribution. A two-way analysis of variance was performed to ascertain the presence of an interaction effect between the group and time factors. If an interaction effect is present, we evaluated whether there are separate effects for time or group. If no interaction effect is detected, we determined if there are any main effects. The *post hoc* comparisons between groups at the same time points were adjusted by Bonferroni to ensure that the overall type I rate was not greater than 0.05 for each analysis of variance. The level of significance was set to α = 0.05. The calculation method for the increase percentage is determined by subtracting the baseline data from Week 8 data, dividing it by the baseline data, and then multiplying it by 100%. Similarly, for calculating the decrease percentage, we subtracted the Week 8 data from the baseline data, divided it by the baseline data, and multiplied it by 100%.

## 3 Results

**FIGURE 2:**
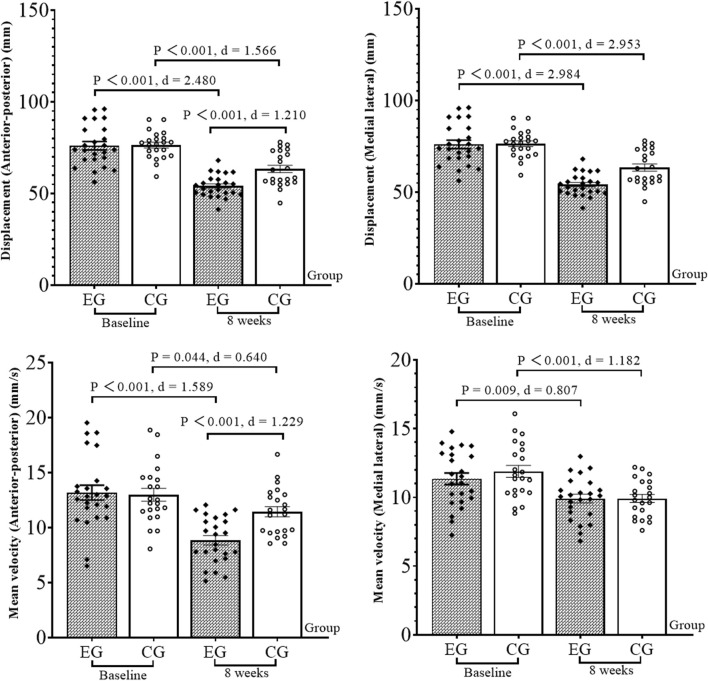
Results of the balance ability test. EG, experimental group; CG, control group; d, Cohen’s d.

**FIGURE 3:**
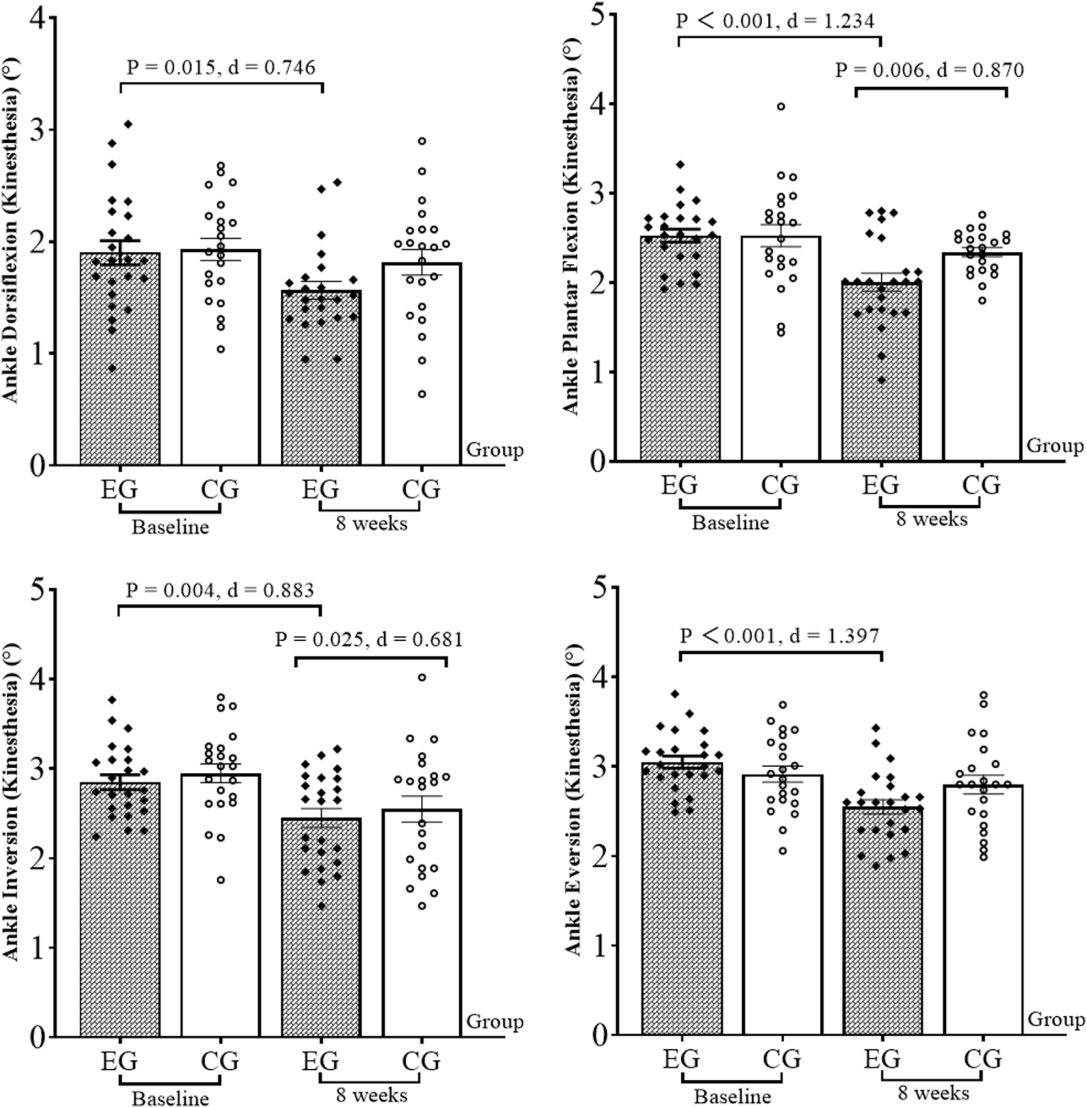
Results of the kinesthetic sensation test. EG, experimental group; CG, control group; d, Cohen’s d.

**FIGURE 4:**
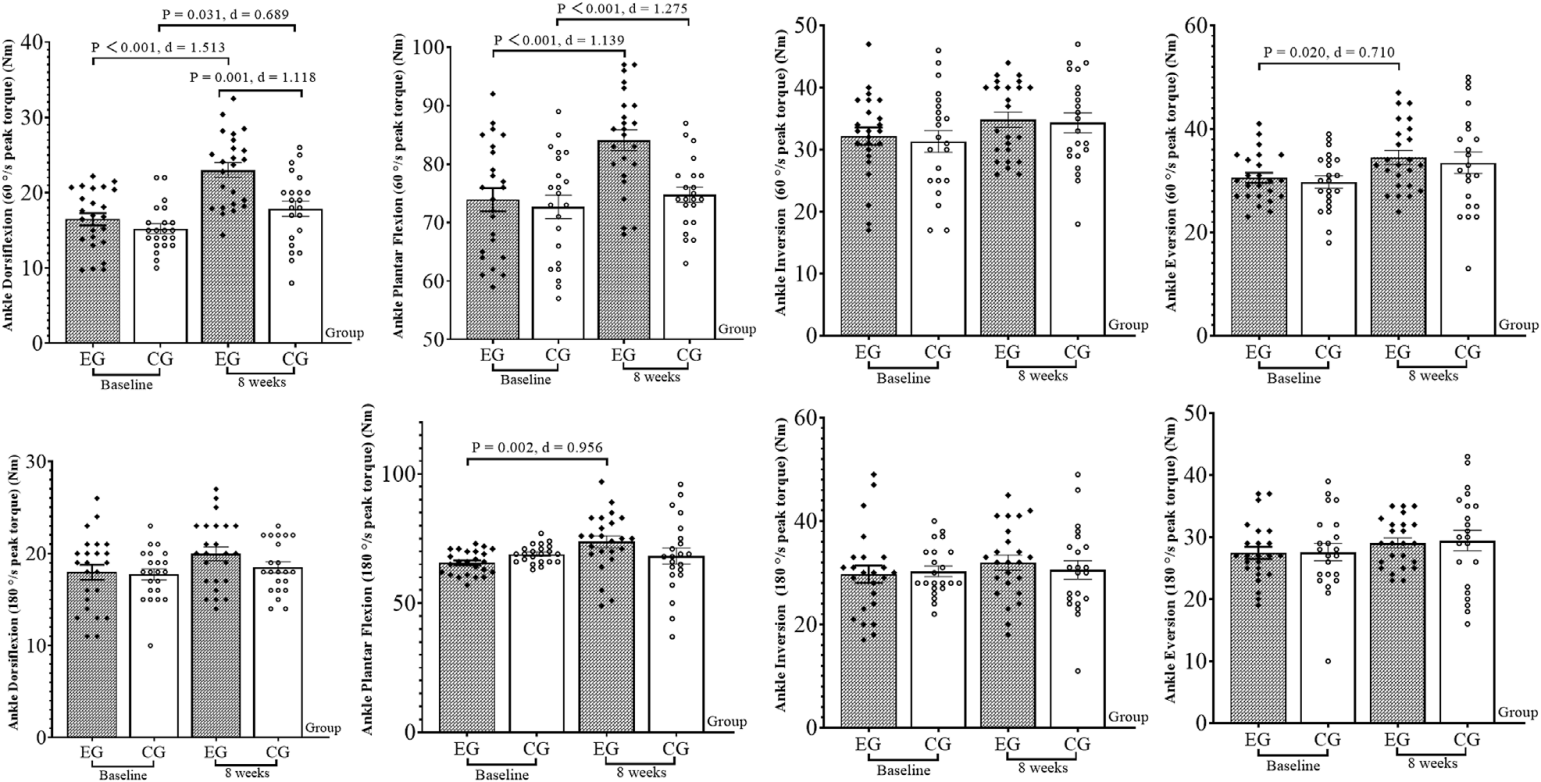
Results of the isokinetic strength test. EG, experimental group; CG, control group; d, Cohen’s d.

The results of the CAIT (Figures 1J), balance ability (Figure 2), ankle kinesthetic sensation (Figure 3), and ankle isokinetic muscle strength (Figure 4) tests in both groups are shown in figures. The normality of the data was first measured using the Shapiro–Wilk test. The results show that all measurements were fit to a normal distribution. No significant difference was observed at baseline between the experimental and control group data (*p* > 0.05).

Ankle dorsiflexion and plantar flex PT (60°/s; F_(3, 88)_ = 4.833, *p* = 0.031; F_(3, 88)_ = 5.085, *p* = 0.027), plantar flex PT (180°/s; F_(3, 88)_ = 4.921, *p* = 0.029), anterior–posterior displacement (F_(3, 88)_ = 5.880, *p* = 0.017), and anterior–posterior mean velocity (F_(3, 88)_ = 6.442, *p* = 0.013) had interactions. A separate effect of group or time was determined. Without the interaction of other indicators, the presence of a main effect was determined. The specific results are as follows:

At 8 weeks, compared with the baseline, the CAIT increased by 13.7% (*p* = 0.014, Cohen’s d = 0.756) in the experimental group. The anterior–posterior and medial–lateral displacement decreased by 28.9% and 31.6% (*p* < 0.001, Cohen’s d = 2.480; *p* < 0.001, Cohen’s d = 2.984), and the anterior–posterior and medial–lateral mean velocity decreased by 33.3% and 12.4% (*p* < 0.001, Cohen’s d = 1.589; *p* = 0.009, Cohen’s d = 0.807), respectively, in the experimental group. Ankle dorsiflexion, plantar flex, and evert PT (60°/s) increased by 39.4%, 13.7%, and 14.2% (*p* < 0.001, Cohen’s d = 1.513; *p* < 0.001, Cohen’s d = 1.139; *p* = 0.020, Cohen’s d = 0.710), respectively, in the experimental group. Plantar flex PT (180°/s) increased by 12.3% (*p* = 0.002, Cohen’s d = 0.956). The dorsiflexion and plantar flex kinesthetic sensation test angles decreased by 17.4% and 20.6% (*p* = 0.015, Cohen’s d = 0.746; *p* < 0.001, Cohen’s d = 1.234), respectively, in the experimental group; for the invert and evert kinesthetic sensation, the test angle reduced by 15.0% and 17.2% (*p* = 0.004, Cohen’s d = 0.883; *p* < 0.001, Cohen’s d = 1.397), respectively. For the control group, CAIT increased by 13.8% (*p* = 0.007, Cohen’s d = 0.883). The anterior–posterior and medial–lateral displacement decreased by 17.1% and 29.4% (*p* < 0.001, Cohen’s d = 1.566; *p* < 0.001, Cohen’s d = 2.593), respectively; anterior–posterior and medial–lateral mean velocity decreased by 12.3% and 16.8% (*p* = 0.044, Cohen’s d = 0.640; *p* < 0.001, Cohen’s d = 1.182), respectively. For the invert kinesthetic sensation, the test angle decreased by 15.2% (*p* = 0.025, Cohen’s d = 0.681). The ankle dorsiflexion PT (60°/s) increased by 17.9% (*p* = 0.031, Cohen’s d = 0.689).

For the comparison between groups, after 8 weeks, the ankle plantar flex kinesthetic sensation test angle (*p* = 0.006, Cohen’s d = 0.870), anterior–posterior displacement (*p* < 0.001, Cohen’s d = 1.210), and anterior–posterior mean velocity (*p* < 0.001, Cohen’s d = 1.229) in the experimental group were significantly lower than those in the control group. The ankle dorsiflexion and plantar flex PT (60°/s) were significantly higher than those in the control group (*p* = 0.001, Cohen’s d = 1.118; *p* < 0.001, Cohen’s d = 1.275).

## 4 Discussion

This study tested some of the study hypotheses. Compared to strength training, strength training alone with additional acupuncture had a better improvement on anterior–posterior balance ability, ankle dorsiflexion, and plantar flex power and plantar flex kinesthetic sensation in CAI participants.

This study showed that both 8 weeks of strength training and strength training with acupuncture increased CAIT scores, indicating a positive effect on both ankle stability. An earlier meta-analysis compared acupuncture with acupuncture without CAI (17 studies, 1,820 patients included) and found acupuncture was more effective than control conditions in relieving pain, thus promoting the return to normal activities and improving the quality of life (Park et al., 2013). Therefore, the combination of strength training with acupuncture has increased CAIT scores compared with strength training. Acupuncture through the stimulation of specific acupoints promotes blood circulation and nerve conduction, thus effectively relieving pain symptoms caused by ankle instability (Huang et al., 2023). It promotes the blood circulation and metabolism of soft tissues and accelerates the repair and regeneration of damaged tissues (de Oliveira and de Freitas, 2016). For ligament or soft tissue injuries caused by CAI, acupuncture may help facilitate recovery.

This study showed that adding acupuncture to strength training could further improve anterior–posterior balance ability in CAI participants. These findings suggest that additional acupuncture effectively improved balance ability anterior–posterior in CAI participants. In a previous study, the static balance ability decreased in CAI participants compared with healthy participants (Abdo et al., 2020). Compared to healthy participants, static balance ability decreased when standing on one leg (Jaffri et al., 2020). After an acupuncture intervention with 32 CAI participants, a significant increase was observed in anterior–posterior and medial–lateral balance (López et al., 2021). Previous finding is similar to this study’s findings. Acupuncture affects the function of the nervous system, adjusts the nerve conduction (López et al., 2021), and helps improve the coordination and stability of the nerves around the ankle. This can improve the perception and response of the ankle and reduce the risk of another sprain.

Proprioception affects the stability and flexibility of human joints, and is the basic for the human body to efficiently complete motor movements, maintain balance, and avoid sports injuries (Cheng et al., 2017). A meta-analysis indicated that CAI caused a decrease in the ankle kinesthetic sensation. CAI caused a decreased invert and evert kinesthetic sensation compared to the healthy group, whereas plantar flex dorsiflexion kinesthetic sensation showed no difference (Ma et al., 2021). This study showed that adding acupuncture to strength training further enhanced the ankle plantar flex kinesthetic sensation, and this finding is different from the previous finding (Ma et al., 2021). This finding may be related to the additional strength training in this study. The previous study observed the difference between the efficacy of acupuncture and physiotherapy on proprioception in CAI athletes, showing acupuncture improved proprioception in athletes and showed better results than traditional physiotherapy (Zhu et al., 2012). Acupuncture is a possible treatment for improving proprioception by changing the muscle’s length–tension relationship and leveraging minor acute discomfort to improve muscle spindle afferent information *via* the gamma motor system (Mullins et al., 2021). In addition, acupuncture improves the function and control of the muscles around the ankle (Zhang et al., 2022) and helps improve the ankle stability and motor control by adjusting the muscle tension and coordination (Yan et al., 2013).

At present, the decrease in human ankle muscle strength caused by CAI is controversial. A meta-analysis of 20 studies (12,397 cases in CAI) found that individuals with CAI have ankle invert and evert strength deficits (Khalaj et al., 2020). However, a study pointed out that ankle plantar flex, dorsiflexion, and invert and evert muscle strength decreased in CAI participants (Santos and Liu, 2008). Wilkerson et al. (1997) believed that decreased invert muscle strength may cause ankle sprain. However, Fox et al. (2008) suggested that decreased plantar flex muscle strength may be related to the altered motor neuron excitability. This study found that a single strength training session only significantly increased the dorsiflexion PT in CAI participants but not in others (plantar flex, and invert and evert). However, strength training with additional acupuncture significantly increased the plantar flex, dorsiflexion, and invert and evert strength. Therefore, strength training along with acupuncture can improve ankle muscle strength in CAI participants (compared to strength training). The possible mechanism is that acupuncture improves the function and control of muscles around the ankle by adjusting muscle tension and coordination to enhance muscle strength (Zhang et al., 2022).

This study has some limitations. No separate acupuncture treatment was involved in the study design, the intervention was not of a limited duration, and participant’s dynamic balance, gait, and neuromuscular response were not measured. These will be the directions of future research.

## 5 Conclusion

Eight weeks of strength training improved CAIT scores, static balance ability, ankle dorsiflexion strength, and invert kinesthetic sensation in CAI college students. Strength training with acupuncture improved the CAIT score, static balance ability, ankle dorsiflexion, plantar flex, invert and evert kinesthetic sensation, and muscle strength. This study shows that adding acupuncture to strength training can further improve the balance ability of anterior–posterior, ankle dorsiflexion, and plantar flex power and plantar flex kinesthetic sensation in CAI among college students.

## Data Availability

The raw data supporting the conclusion of this article will be made available by the authors, without undue reservation.
